# Ketamine and neuroticism: a double-hit hypothesis of internalizing disorders

**DOI:** 10.1017/pen.2020.2

**Published:** 2020-03-19

**Authors:** N. McNaughton, P. Glue

**Affiliations:** 1 Department of Psychology, University of Otago, Dunedin, New Zealand; 2 Department Psychological Medicine, University of Otago, Dunedin, New Zealand

**Keywords:** Ketamine, Neuroticism, Internalizing disorders, Anxiety, Depression, Post-traumatic stress disorder

## Abstract

Psychiatric disorders can often be viewed as extremes of personality traits. The primary action of drugs that ameliorate these disorders may, thus, be to alter the patient’s position on a relevant trait dimension. Here, we suggest that interactions between such trait dimensions may also be important for disorder. Internalizing disorders show important differences in terms of range of activity and speed of response of medications. Established antidepressant and anxiolytic medications are slow in onset and have differing effects across different internalizing disorders. In contrast, low-dose ketamine is rapidly effective and improves symptom ratings in all internalizing disorders. To account for this, we propose a “double hit” model for internalizing disorders: generation (and maintenance) require two distinct forms of neural dysfunction to coincide. One hit, sensitive to ketamine, is disorder-general: dysfunction of a neural system linked to high levels of the personality trait of neuroticism. The other hit is disorder-specific: dysfunction of one of a set of disorder-specific neural modules, each with its own particular pattern of sensitivity to conventional drugs. Our hypothesis applies only to internalizing disorders. So, we predict that ketamine will be effective in simple phobia and (perhaps partially) in anorexia nervosa, but would make no such prediction about other disorders where neuroticism might also be important secondarily (e.g. attention deficit hyperactivity disorder and schizophrenia).

Factor analysis of common mental disorders identifies two main groups: internalizing and externalizing disorders (DeYoung & Krueger, 2018; Krueger, McGue & Iacono, 2001; Slade & Watson, 2006). Internalizing disorders, characterized by quiet, internal distress, include DSM-5 (American Psychiatric Association, 2013) diagnoses such as generalized anxiety disorder (GAD), social anxiety disorder (SAD, previously social phobia), major depressive disorder (MDD), panic disorder (PD), post-traumatic stress disorder (PTSD), obsessive compulsive disorder (OCD), and simple phobia (SP). Drug treatment of GAD, SAD, SP, PD, OCD, MDD, and PTSD has identified an apparently paradoxical pattern of treatment responses. In this article, we will first outline the paradox and then suggest a novel hypothesis as a solution.

## 1. The paradox

On the one hand, internalizing disorders have different response patterns to a range of slow-acting conventional medications (Table 1). In particular, “anxiolytic” drugs do not affect SP, PD, OCD, MDD, or PTSD (which essentially combines these other disorders). This disparate pattern of drug class effectiveness suggests that these different DSM diagnoses involve dysfunction of different neural systems. Equally, none of the drugs are completely effective in any disorder suggesting that the DSM classes need modification, and it is argued below that the true underlying disorders should be seen as the result of extremes of traits.

**Table 1.** Comparison of the effects of different drug classes on internalizing disorders. Drugs are ordered in approximate relation to general effectiveness from most effective to least effective (or least available data)

	GAD	SAD	MDD	PD	PTSD	OCD	SP
Ketamine	+	+	+	+	+	+	
Mirtazapine	+	+	+	+	+	+	
SSRI	+	+	+	+	+	++	(+)
SNRI	+	+	+	+	+	+	
TCA_2_ (≈CMI)	+	(+)	+	++	(+)	++	
TCA_1_ (≈IMI)	+	(+)	+	+	+	(+)	0
MAOIs		+	+	+	(+)	(+)	(+)
NRI		(+)	+	(+)	(+)		
Buspirone	+	(+)	+	0	0	(+)	
BDZ_2_	+	(+)	(+)	(+)	0	0	0
BDZ_1_	+	(+)	0	0	0	0	0
Pregabalin	+	+	0*	(0)			

Disorder abbreviations: GAD, generalized anxiety disorder; MDD, major depressive episode; OCD, obsessive-compulsive disorder; PD, panic disorder; PTSD, post-traumatic stress disorder; SAD, social anxiety disorder (previously social phobia); SP, simple phobia. Activity codes: blank, no data; 0, inactive; (0), equivocal inactivity; (+), equivocal or high dose activity; +, active; ++, more strongly active than related drugs.

BDZ_1_, early benzodiazepines, for example, chlordiazepoxide and diazepam administered at typical (i.e. low) antianxiety doses. Other sedative antianxiety drugs (barbiturates, meprobamate) have similar effects; BDZ_2_, later high potency benzodiazepines, for example, alprazolam. The antipanic effect is achieved at higher doses and this has also been reported with equivalent high doses for BDZ_1_ (Noyes et al., 1996). See also van Marwijk, Allick, Wegman, Bax and Riphagen (2012); MAOI, monoamine oxidase inhibitors, for example, phenelzine; SSRI, selective serotonin reuptake inhibitors, for example, fluoxetine, citalopram; TCA_2_, high effect tricyclics, for example, clomipramine; TCA_1_, imipramine and related tricyclic antidepressants, but excluding clomipramine.

*PG unpublished results, Hauser, Petzke and Sommer (2010), but see also discussion in Strawn and Geracioti (2007).

Updated from McNaughton and Zangrossi (2008) data from or as reviewed in Albucher and Liberzon (2002), Atmaca, Tezcan and Kuloglu (2003) den Boer and Westenberg, 1988, Feltner, Liu-Dumaw, Schweizer and Bielski (2011), Gray and McNaughton (2000), McNaughton (2002), Pande et al. (2004), Ravindran, Kim, Letamendi and Stein (2009), Ravindran & Stein (2010), Rickels and Rynn (2002), Spivak et al. (2006), Stein, Hollander, Mullen, DeCaria and Liebowitz (1992), Stein, Vythilingum and Seedat (2004), Stevens and Pollack (2005), Westenberg (1999).

On the other hand, internalizing disorders share a common risk factor of neuroticism. Andrews and colleagues stated “the lifetime experience of more than one diagnosis of a neurotic syndrome was common but there was no evidence of patterns of co-occurrence of diagnoses being associated with particular syndromes” (Andrews, Stewart, Morris-Yates, Holt & Henderson, 1990, p. 6). In one “study, the correlation between internalizing psychopathology and neuroticism approached 1.0, suggesting that neuroticism may be the core of internalizing psychopathology” (Griffith et al., 2010, p. 1125). Importantly, all these disorders appear at least partially sensitive to *long-term* treatment with at least some specific serotonin reuptake inhibitors (SSRIs; Table 1). Of particular note, for all of the internalizing disorders that have so far been tested – and which span multiple nominal classes whether from the perspective of ICD-10 (World Health Organization, 1992, 2010) or DSM-5 – ketamine has been found to be rapidly and broadly effective in cases resistant to conventional drugs, including the SSRIs (Feder et al., 2014; Glue et al., 2017; Loo et al., 2016; Rodriguez et al., 2013; Zarate Jr et al., 2006; Zhang & Hashimoto, 2018) – creating what has been termed “a paradigm shift for depression research and treatment” (Krystal, Abdallah, Sanacora, Charney & Duman, 2019). This common fast response to ketamine suggests that the disorders involve dysfunction of a single common neural system.

## 2. A resolution of the paradox

Here, we resolve the multi- versus single-system paradox presented by these treatment results with the suggestion that these disorders involve a “double hit” – two distinct forms of neural extreme must coincide to generate (or maintain) any internalizing disorder. We propose that, for one of these hits, high sensitivity of neural systems supporting neuroticism and sensitive to ketamine provides not only risk for (as in high blood pressure creating a risk for stroke) but also a necessary ongoing component of all these internalizing disorders. That is, the presence of high neuroticism alone represents only a risk factor for neurotic disorders not a form of psychiatric disorder in itself. For the other hit, a disorder-specific system, with its own particular pattern of sensitivity to conventional drugs, provides a second necessary component (or several such systems do so where there is comorbidity). Thus, neuroticism, rather than simply precipitating, say, high trait anxiety (which would then by itself constitute disorder) is required to be compounded with high trait anxiety (and vice versa) to generate disorder. Similarly, high trait anxiety might require the addition of an increase in neuroticism to generate disorder.

This shared+unique perspective on specific neural causes is in some respects similar to the symptom-factor-based tripartite (shared general distress, anxiety-specific hyperarousal, and depression-specific anhedonia) model of Clark and Watson (1991), the hierarchical model of Zinbarg and Barlow (1996), and the combination of these two models into “an integrative hierarchical model of anxiety and depression” (Mineka, Watson & Clark, 1998). It differs in: (1) treating neuroticism as primary - as opposed, like anxiety and depression, to simply being a consequence of “a shared genetic factor that reflects general individual differences in subjective distress and negative affectivity” (Mineka et al., 1998, p. 391); (2) being explicitly about an interaction of *neural causes* that constitute distinct therapeutic targets rather than just symptom overlap; (3) in explicitly including specific phobia, panic, OCD, and social anxiety; and (4) in explicitly excluding personality and conduct disorders, and schizophrenia (compare Mineka et al., 1998, pp. 397–398).

**Figure 1.**
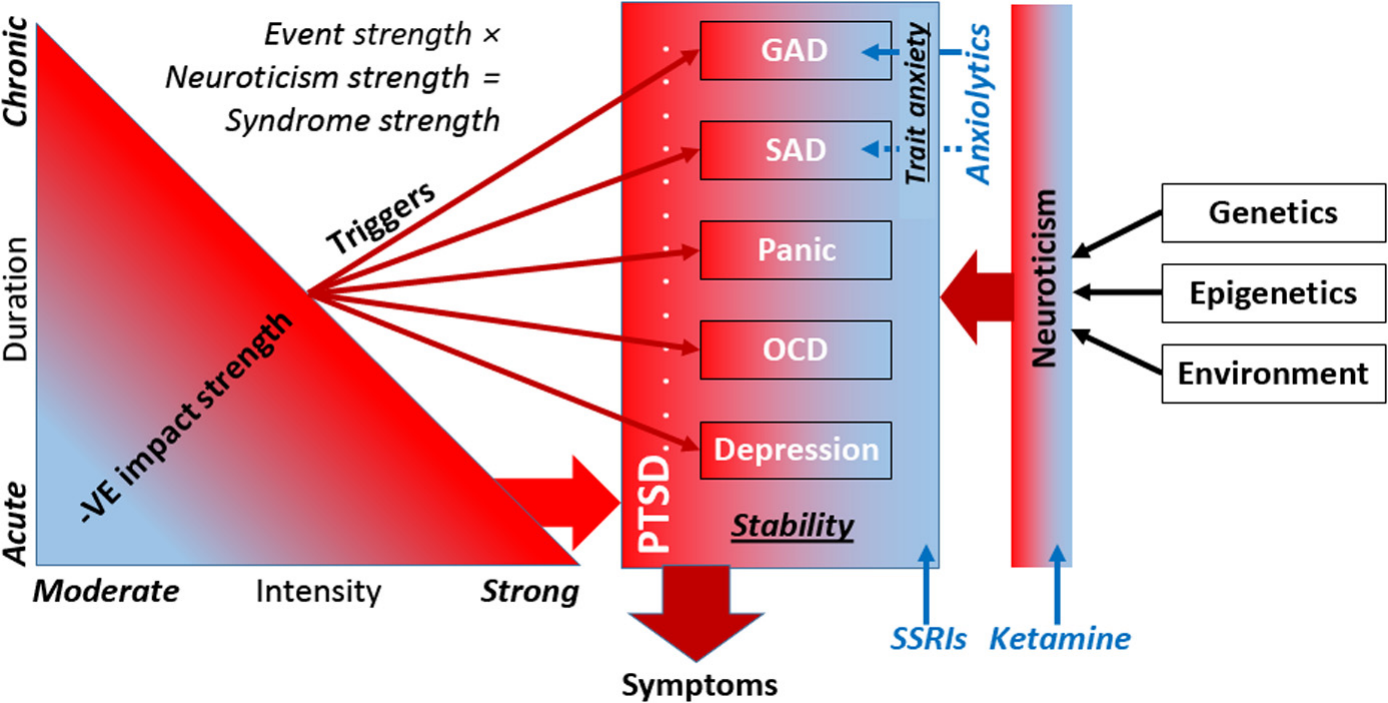
Diagrammatic representation of the 2-hit hypothesis for disorders known to be affected by ketamine. Disorder is held to result when high neuroticism (resulting from genetic, epigenetic, and prior environmental factors) is combined with a high level of a specific trait linked to GAD, SAD, panic, OCD, or depression. High levels of these specific traits can be triggered by moderate chronic or strong acute stress. In the latter case PTSD may result. Specific anxiolytic drugs affect the specific trait linked to GAD and to a lesser extent SAD (see Table 1). SSRIs act on a background higher order trait of “stability” (DeYoung, 2006), which would not be specific to aversive events (Carver et al., 2008) and which would slowly alter the specific trait levels. Ketamine acts to affect neuroticism and so alters the disordered expression of all the specific traits.

Our double-hit resolution of the paradox is presented schematically in Figure 1. It combines the effects of usually pre-existing neuroticism with the impact of traumatic or chronically stressful events on specific systems that control particular symptoms and can provide the basis for particular syndromes.

## 3. The role of neuroticism – Hit 1 (disorder-general)

Shown on the right-hand side of Figure 1, genetics (Anttila et al., 2018; Luciano et al., 2018), epigenetics, and previous environmental influences determine the current level of neuroticism (Hecht, Van Calker, Berger & Von Zerssen, 1998; Ormel, Oldehinkel & Brilman, 2001). Neuroticism can be viewed as a neural sensitivity that is the basis for “relatively stable tendencies to respond with negative emotions to threat, frustration, or loss. *Individuals in the population vary markedly on this trait*, ranging from frequent and intense emotional reactions to minor challenges to little emotional reaction even in the face of significant difficulties” (Lahey, 2009, p 241, our emphasis). Strong links can be drawn between major personality dimensions and psychopathology (DeYoung & Krueger, 2018), and, in particular, high neuroticism is a common dimension for internalizing disorders (Griffith et al., 2010) and “in the general population, the personality trait neuroticism is significantly correlated with almost every psychiatric disorder and migraine” (Anttila et al., 2018, p. 1). As a result, neuroticism is coming to be seen as of immense importance to public health (Lahey, 2009; Widiger & Oltmanns, 2017).

Our use of “neuroticism” as a surface label for the sensitivity of the key set of neural systems acted on by ketamine involves a degree of approximation. Neuroticism, as the term is currently used, has been reliably extracted as a factor in analysis of questionnaires and it forms a key component of the “Big Five” consensus on personality structure (DeYoung, Weisberg, Quilty & Peterson, 2012; McAdams & Pals, 2006), but its derivation is largely lexical and, while there are indications as to its neural basis, its neurology is not well established (DeYoung et al., 2010; Ormel et al., 2013; Servaas et al., 2013) and, as usually extracted, it “is difficult to distinguish statistically from the general risk factor for the *internalizing* disorders” (DeYoung & Krueger, 2018, p. 27; Griffith et al., 2010).

We would emphasize that in Figure 1 “neuroticism” is intended to refer to the *variation in the population of the sensitivity* of a set of, as yet undetermined, phylogenetically old neural systems (on which a single dose of ketamine has rapid-onset effects that last approximately 1 week), and that identification of this neural basis could ultimately redefine the current lexicon-based concept (McNaughton & Smillie, 2018).

### 3.1 Neural targets of ketamine

Even less definite, are the systems through which ketamine is generating its therapeutic effects on disorders that are resistant to other treatments. Ketamine is, among other things, an N-methyl-D-aspartate receptor (NMDAR) antagonist developed in the 1960 s as an anaesthetic (see Domino, 2010). In their comprehensive review of ketamine’s pharmacology, Zanos and Gould noted that “clinical trials indicate that … alternative NMDAR antagonists lack the rapid, robust and/or long-lasting antidepressant actions of ketamine in humans” (Zanos & Gould, 2018, p. 804). This suggests that ketamine’s therapeutic effect may only partly involve NMDAR antagonism or may perhaps not involve the NMDAR at all.

Ketamine’s actions are complex. It is scheduled because of abuse liability but has been approved for use as a paediatric anaesthetic and is also used off-label for pain and sedation. Importantly, effective doses for treating internalizing disorders appear to occupy a low and narrow range – well below the anaesthetic level and narrower than the range used for pain relief (Glue, Gulati, Le Nedelec & Duffull, 2011). Likewise, it has broad effects (including both increases and decreases in power) across a wide range of electroencephalographic bands and electrodes, but its therapeutic action correlates with change in only a narrow band at a restricted location (Shadli et al., 2018).

### 3.2 Neuroticism, serotonin, and stability

In this context, we also need to distinguish the “neurotic” systems from serotonergic ones. SSRIs, tricyclic antidepressants, and 5HT_1A_ partial agonists like buspirone all act via serotonin (5HT) systems that provide widespread innervation of the neocortex. But, the different classes of drug have impacts on the different neurotic disorders that vary qualitatively or quantitatively depending on variation in receptor and uptake system subtypes in different parts of the brain.

While 5HT innervation includes the defense systems that are thought to support neurotic disorders (McNaughton & Corr, 2004, 2016), the role of 5HT appears much broader (Carver, Johnson & Joormann, 2008), and in personality terms appears linked to the higher order meta-trait of stability within which neuroticism is just one domain (DeYoung, 2006).

Critically, the effects of serotonergic drugs are slow rather than fast and ketamine is quickly effective in those who are resistant to them. In Figure 1, therefore, we show serotonin as modulating all the key specific trait systems linked to specific disorders and not acting directly on the more general factor of neuroticism. This serotonergic modulation of the specific defense systems could involve different aspects of the 5HT system (different source cells, different receptor system sensitivities, and different levels of reuptake) and so contribute to different disorders under different circumstances.

## 4. The role of other factors

### 4.1 Disorder-specific traits – Hit 2

Another important distinction in Figure 1 is of neuroticism from trait anxiety. Low potency benzodiazepines, buspirone, and pregabalin share only anxiolytic action and not panicolytic, antidepressant, or other effects.

Comparison of the actions of these anxiolytics across a wide range of behaviours and neurophysiology has allowed a distinction between anxiety (as defensive approach) and fear (as defensive avoidance) and has mapped the two to distinct neural systems (Gray & McNaughton, 2000; McNaughton & Corr, 2004).

In turn, this neural model has provided the basis for development of the Reinforcement Sensitivity Theory of personality (Corr, 2008; McNaughton & Corr, 2008) in which the drugs (and by implication equivalent endogenous ligands) are held to reduce the effects of a form of trait anxiety. In the sense defined by this theory, which is more restricted than that of the commonly used Spielberger State-Trait Anxiety Inventory (Spielberger, Gorusch, Lushene, Vagg & Jacobs, 1983), trait anxiety would be a specific factor contributing to what DSM classifies as GAD and, to some extent, SAD (Figure 1). It would not contribute to other DSM internalizing disorders (SP, PD, OCD, and MDD) – which would depend on some form of trait phobia, trait panic, trait obsession, and trait depression, respectively. (We consider PTSD as involving multiple such traits, see below.) Importantly, although it is convenient to talk about disorders and their targeting by drugs in terms of DSM diagnostic classes, we would see our approach to disorder as aligning more with a dimensional spectrum approach (Kotov, Krueger & Watson, 2018; Kotov et al., 2017; Widiger et al., 2018).

In all cases of internalizing disorder, we propose that the high levels of the specific trait (such as trait anxiety) must be combined with high levels of neuroticism to generate the specific disorder. Within the Big Five system, this would make trait anxiety an aspect or even a lower order facet (see Figure 2 in DeYoung & Krueger, 2018) of neuroticism – with the two having substantially correlated sensitivities in the general population but depending on quite distinct, but potentially interacting, neural systems within the individual.

### 4.2 Life events

Strong acute (trauma) or chronic moderate (stress) events can account for the elicitation of both more specific and more general (comorbid) patterns of disorder (left-hand side of Figure 1). The effects of events are most obvious with the strong acute ones that are the basis for a diagnosis of PTSD, which can then involve a wide variety of comorbidities. While trauma is, by definition, necessary for PTSD, it is not sufficient on its own. Interestingly, in Australian male fire fighters “neuroticism and a past history of treatment for a psychological disorder were better predictors of post traumatic morbidity than the degree of exposure to the disaster or the losses sustained. These results raise doubts about the postulated central aetiological role a traumatic event plays in the onset of morbidity” (McFarlane, 1989, p. 221). In this Australian study, neuroticism was measured after the event and so could have been affected by it (Jeronimus, Ormel, Aleman, Penninx & Riese, 2013) but this cannot easily account for the lack of relation between the level of traumatic exposure and the occurrence of PTSD.

Similarly, while experiencing an internalizing disorder predicts further internalizing disorders in general, it does not predict recurrence of the specific disorder initially experienced (Andrews et al., 1990). In Figure 1, therefore, we present a picture of a variety of events capable of triggering occurrence of a variety of internalizing disorders. With chronic events, such triggers would interact with learning and autonomic systems to maintain morbidity and generate comorbidity (McNaughton, 2008; McNaughton & Corr, 2016) or, through sheer repetition, lead to kindling (Adamec & Young, 2000; Kellett & Kokkinidis, 2004). Note that while we focus on triggers as inputs to specific systems to elicit specific disorders, we include “environment” as an input to neuroticism – where the long-term accumulation of positive and negative life events can adjust sensitivity (Jeronimus et al., 2013).

### 4.3 Exclusion of externalizing disorders

Neuroticism has a high genetic correlation with anxiety disorders and MDD, and weaker, but still consistent, relationships with most other psychiatric disorders (Anttila et al., 2018). At present, we would see our two-hit hypothesis as applying to the former (anxiety/phobia/MDD) more than the latter (in contrast to the tripartite model and its variants, Mineka et al., 1998).

In the weakly correlated cases (e.g. schizophrenia) the importance of neuroticism may not be primarily as a pre-existing risk factor for the disorder itself. Neuroticism may have a more secondary impact on the capacity for stressors to impact on the disorder (Fowles, 1992; Gispen-de Wied, 2000). Neuroticism could also be involved because of links between the disorder (e.g. ADHD) and a predisposition to anxiety disorders (Anttila et al., 2018), with which neuroticism would then interact. Importantly, with neither schizophrenia nor ADHD (which both have externalizing features) is there an apparently indiscriminate transfer from one disorder to the other of the sort seen with neurotic disorders (Andrews et al., 1990), nor does either commonly transfer to any of the internalizing disorders. (By transfer, we mean remission from one disorder before onset of the next and exclude comorbidity.) We have no reason, therefore, to group this weakly correlated class of disorders with the class focussed on by Andrews et al. (1990), which is strongly linked to neuroticism and where each disorder is strongly linked to the others over an individual’s life.

## 5. Predictions from the model

Based on our model, we would expect ketamine to demonstrate activity in simple phobia and on the affective and compulsive aspects of anorexia nervosa. There are preliminary positive open-label data for the latter patient group (Drumm et al., 2014; Mills, Park, Manara & Merriman, 1998).

We would not expect it to be directly effective in externalizing disorders. It might be effective in treating comorbid anxiety or depression in ADHD or schizophrenia, respectively. But, while improvement of the comorbid internalizing disorder could indirectly alter the course of the primary disorder, we would not expect ketamine to have a direct effect and we are certainly not suggesting that its use would be advisable.

Conversely, the lack of effectiveness of ketamine in augmenting ECT’s antidepressant effects (McGirr et al., 2017) could reflect the lower neuroticism scores in patients with melancholic depression (Kendler, 1997), who may be more likely to be treated with ECT than patients with non-melancholic depression. Assessing the impact of neuroticism score on response to augmentation could clarify if there is a subgroup of patients receiving ECT who might gain additional benefit from ketamine.

Neuroticism is also genetically linked to disorders such as migraine and Tourette syndrome. However, we have no reason to group these with our current set of neurotic disorders (Table 1).

## 6. Conclusions

In sum, for those internalizing disorders where neuroticism has been shown to be a predisposing factor, and where there is often progression from one disorder to another, we propose a two-hit hypothesis of disorder: both trait neuroticism and a trait linked to the specific disorder must be high. Disorder-specific treatments act on neural systems underlying the specific trait, while ketamine acts on neural systems underlying neuroticism. While neuroticism can be important for many mental disorders, we propose that those involving the proposed double hit represent a specific “internalizing disorder” class. Should our positive and negative predictions prove accurate, this would suggest that all these disorders could be included into a single diagnostic cluster rather than the multiple, somewhat different, clusters proposed by DSM-5 and ICD-10. On this view, there would be a hierarchical organization of the internalizing disorders with a common single “parsimonious” internalizing factor and subordinate aspects distinguishing distress disorders and fear disorders with likely even finer structure facets linked to, for example, trait anxiety as we have defined it (DeYoung & Krueger, 2018; Seeley, Kosty, Farmer & Lewinsohn, 2011).

## Research Transparency and Reproducibility

This paper is a review article and so there are no data or methods not already in the public domain.
